# Exome sequencing in undiagnosed inherited and sporadic ataxias

**DOI:** 10.1093/brain/awu348

**Published:** 2014-12-13

**Authors:** Angela Pyle, Tania Smertenko, David Bargiela, Helen Griffin, Jennifer Duff, Marie Appleton, Konstantinos Douroudis, Gerald Pfeffer, Mauro Santibanez-Koref, Gail Eglon, Patrick Yu-Wai-Man, Venkateswaran Ramesh, Rita Horvath, Patrick F. Chinnery

**Affiliations:** 1 Wellcome Trust Centre for Mitochondrial Research, Institute of Genetic Medicine, Newcastle University, Central Parkway, Newcastle upon Tyne, NE1 3BZ, UK; 2 Department of Clinical Biochemistry, Royal Victoria Infirmary, Newcastle upon Tyne Foundation Hospitals NHS Trust, Newcastle upon Tyne, NE1 4LP, UK; 3 Department of Paediatric Neurology, Royal Victoria Infirmary, Newcastle upon Tyne Foundation Hospitals NHS Trust, Newcastle upon Tyne, NE1 4LP, UK

**Keywords:** ataxia, whole exome sequencing

## Abstract

Inherited ataxias are clinically and genetically heterogeneous, and a molecular diagnosis is not possible in most patients. Having excluded common sporadic, inherited and metabolic causes, we used an unbiased whole exome sequencing approach in 35 affected individuals, from 22 randomly selected families of white European descent. We defined the likely molecular diagnosis in 14 of 22 families (64%). This revealed *de novo* dominant mutations, validated disease genes previously described in isolated families, and broadened the clinical phenotype of known disease genes. The diagnostic yield was the same in both young and older-onset patients, including sporadic cases. We have demonstrated the impact of exome sequencing in a group of patients notoriously difficult to diagnose genetically. This has important implications for genetic counselling and diagnostic service provision.

## Abstract (precis)

Inherited ataxias are difficult to diagnose genetically. Pyle *et al.* use whole-exome sequencing to provide a likely molecular diagnosis in 14 of 22 families with ataxia. The approach reveals *de novo* mutations, broadens the phenotype of other disease genes, and is equally effective in young and older-onset patients.

## Introduction

The inherited ataxias are clinically and genetically heterogeneous, presenting at any age, and usually without features pointing to a specific molecular diagnosis (Anheim *et al.*, 2010; Jayadev and Bird, 2013). Defining the genetic aetiology is important because clinically similar disorders can have different recurrence risks, and in some instances there are treatment implications (Jayadev and Bird, 2013).

The first step in investigating patients is the exclusion of structural, toxic and inflammatory disorders. Following this, testing for the more common trinucleotide repeat neurometabolic disorders provides a diagnosis in up to ∼60% of familial cases. After excluding the more common genetic causes, over 40 different loci are implicated. Subsequent investigation usually proceeds on a gene-by-gene basis, which takes considerable time, and is expensive. Many patients do not receive a genetic diagnosis for many years, if at all, limiting genetic counselling and prenatal diagnosis.

Targeted next-generation sequencing panels have been shown to increase the diagnostic yield in suspected inherited ataxia, particularly in those with adolescent onset and a family history (Hoischen *et al.*, 2010). However, after excluding common forms of spinocerebellar ataxia (SCA) the overall detection rate remains <20% in routine clinical practice (Nemeth *et al.*, 2013). This may be due to the limited portfolio of genes included on custom-designed platforms (Nemeth *et al.*, 2013), but the clinical overlap of different neurogenetic syndromes presents a further challenge. In some patients, ataxia may develop in conjunction with spastic paraplegia or a neuropathy, and the initial clinical presentation may mislead the clinician to select the wrong multi-gene panel. Not based on any prior assumption about the underlying gene defect, whole exome sequencing provides a potential solution to these problems. Here we set out to determine the impact of whole exome sequencing in patients with suspected inherited ataxia who did not have one of the more common forms diagnosed on routine genetic testing.

## Materials and methods

### Inclusion criteria

We studied 35 individuals with unexplained ataxia identified at random through routine referrals to the regional neurogenetics service at Newcastle upon Tyne, England. The clinic serves a region of ∼3 million in the north of England. To ensure that the cohort reflected standard clinical practice, we did not select cases based on age, gender or the presence of a family history. Prior to inclusion, all had routine clinical investigations to exclude treatable causes of acquired ataxia, including brain MRI and CSF examination with oligoclonal band analysis. All had negative genetic testing for SCA1, 2, 3, 6, 7, 17 (now known as *ATXN1*, *ATXN2*, *CACNA1A*, *ATXN7*, and *TBP*, respectively), DRPLA (now known as *ATN1*), FA (now known as *FXN*) and *FMR1* in adult males, which are positive in 17% of routine referrals to our clinic.

### Demographic and clinical characteristics

The 35 affected individuals were from 22 families of white European descent. The mean age was 25 years [standard deviation (SD) 14, range 3–57 years] at the time of the study, and 14 were male. Detailed clinical features and the results of clinical investigations are shown in Table 1 and the Supplementary material. Twenty-five of 35 individuals had a family history, suggestive of dominant inheritance in 11/25, and autosomal recessive in 14/25. There was no known consanguinity.

**Table 1:** Clinical presentation, laboratory investigations and exome sequencing results for 35 patients with unexplained ataxia

ID, Sex	Age of onset (y)	Family history	Symptom at onset	Clinical signs						Investigations					Gene	Variants
				Cerebellar a ataxia	Eyes				Cerebellar dysarthria	Electrophysiology			MRI			
					Jerky ocular pursuit	Gaze evoked nystagmus	Hypometric saccades	Optic atrophy		Axonal sensorimotor neuropathy	Demyelinating sensorimotor neuropathy	Normal	Cerebellar atrophy	Generalized atrophy		
**Confirmed pathogenic**																
P1, M	Teens	Aff sib (P2)	Learning difficulties	**+**	−	−	−	−	−	**+**	**+**	−	**+**	−	*SACS*	c.2076delG:p.Thr692Thr fs*713; c.3965_3966delAC:p. Gly1322Valfs*1343^s^
P2, F	26	Aff sib (P1)	Upper limb clumsiness	**+**	**+**	−	−	−	**+**	**+**	**+**	−	**+**	**+**		

P3, M	Teens	Aff sib (P4)	Upper limb clumsiness	**+**	**+**	**+**	**+**	**+**	**+**	−	−	**+**	−	−	*SACS*	hemizygous c.13048G>T: p.Glu4350*; 0.7Mb deletion on Chr13q12.12^s^
P4, M	Childhood	Aff sib (P3)	Walking delay	**+**	**+**	**+**	−	**+**	**+**	−	−	**+**	**+**	**+**		

P5, M	40	Aff sib (P6)	Gait disturbance	**+**	−	−	−	−	−	−	**+**	−	−	**+**	*SACS*	c.1580C>G:p.Ser527* c.6781C>A:p.Leu2261Ile^s^
P6, F	40s	Aff sib (P5)	Gait disturbance	**+**	**+**	−	−	−	**+**	−	−	−	−	−		

P7, F	23	Aff sib (P8)	Speech, balance	**+**	**+**	−	−	**+**	−	−	−	−	**+**	−	*KCNC3*	het. c.1259G>A:p.Arg420His^s^
P8, F	57	Aff sib (P7)	Upper limb clumsiness	**+**	**+**	−	−	**+**	−	−	−	−	**+**	−		

P9, M	30	Aff sib (P10)	Upper limb clumsiness	**+**	**+**	**+**	−	**+**	**+**	−	−	**+**	−	**+**	*SPG7*	c.1529C>T:p.Ala510Val; c.1715C>T:p.Ala572Val
P10, F	29	Aff sib (P9)	Upper limb clumsiness	**+**	−	**+**	−	−	**+**	−	−	**+**	**+**	−		

P11, F	Infancy	Aff sib (P12), mother (P13)	Delayed motor and speech development	**+**	−	**+**	−	**+**	**+**	−	−	−	**+**	−	*TUBB4A*	het c.900C>A:p.Met300Ile^s^
P12, F	Infancy	Aff sib (P11), mother (P13)	Delayed motor and speech development	**+**	−	**+**	**+**	**+**	**+**	−	−	−	**+**	−		
P13, F	30	Aff children (P11, P12)	Gait disturbance, slurred speech	**+**	−	**+**	−	**+**	**+**	−	−	**+**	**+**	−		mosaic c.900C>A:p.Met300Ile^s^

P14, F	5	None	Learning difficulties, gait disturbance	**+**	−	−	**+**	−	**+**	−	−	**+**	−	−	*TUBB4A*	het c.1091C>A:p.Ala364Asp^s^

P15, F	21	2 Aff sibs (P17)	Gait disturbance	**+**	−	−	**+**	−	+	−	−	**+**	−	−	*NPC1*	c.467T>C: p.Met156Thr c.709C>T:p.Pro237Ser^s^
P16, F	21	2 Aff sibs (P16)	Upper limb clumsiness	**+**	**+**	−	**+**	−	+	−	−	**+**	−	−		

P17, M	30	Aff mother, aff cousin (P19)	Speech disturbance Upper limb clumsiness	**+**	**+**	−	**+**	−	**+**	−	−	−	−	−	*SLC1A3*	het c.1361G>A:p.Arg454Gln^s^
P18, F	39	Aff cousin (P18), aff aunt	Speech disturbance	**+**	**+**	−	−	−	**+**	−	−	−	−	−		

**Possibly pathogenic**																
P19, M	32	None	Gait disturbance	**+**	−	−	−	−	−	**+**	−	−	−	**+**	*ZFYVE26*	c.2338C>T:p.Arg780*
																c.2450delT:p.Leu817Cysfs*12

P20, F	40	None	Gait disturbance	**+**	−	−	−	**+**	**+**	−	−	−	−	−	*WFS1*	c.577A>C:p.Lys193Gln; c.1367G>A:p.Arg456His

P21, F	Infancy	None	Walking delay	+	−	+	−	+	+	−	−	−	−	−	*FASTKD2*	c.-66A>G; c.149A >G:p.Lys50Arg^s^

P22, M	37	Aff son (P23)	Upper limb clumsiness	+	−	+	−	−	+	−	−	−	**+**	−	*ZFYVE27*	c.805-2A>G^s^
P23, M	35	Aff father (P22)		+	−	−	−	−	+	−	−	+	**+**	−		
P24, M	25	Aff father (P22) Aff half-brother (P23)		+	+	+	+	−	+	−	−	−	**+**	−		

P25, F	11	None	Upper limb clumsiness	+	−	+	−	−	+	−	−	+	−	−	*WNK1*	c.1994C>T:p.Thr665Ile^s^ c.3272C>T:p.Thr1091Ile^s^
**Uncertain significance or no candidate variants found**																
P26, F	13	None	Upper limb clumsiness	+	+	+	+	−	+	−	−	−	−	−	*KCNB2*	c.1589C>T: p.Ser530Phe; c.2351T>C:p.Leu784Pro

P27, M	20	2 Aff children	Gait disturbance	+	+	+	−	−	+	−	−	−	+	−	*ABCB7*	hom (hemi) c.818G >A:p.Arg273Gln^s^
															*POLG*	
															*WFS1*	c.2243G>C:p.Trp748Ser; c.3428A>G:p.Glu1143Gly
																het c.1294C>G:p.Leu432Val

P28, F	Childhood	Aff cousin	Gait disturbance	+	+	+	−	−	−	−	−	−	+	−	No candidate variants	
P29, F	40	Aff child (P30)	Gait disturbance	+	+	+	−	−	−	−	−	−	−	+	No candidate variants	
P30, M	21	Aff mother (P29)	Gait disturbance	+	+	−	−	−	−	+	−	−	+	−	No candidate variants	
P31, F	46	None	Gait disturbance	+	+	−	−	−	+	−	−	−	−	−	No candidate variants	
P32, M	31	None	Upper limb clumsiness	+	+	+	−	−	+	−	−	−	+	+	No candidate variants	
P33, M	Teens	Aff sib (P34)	Upper limb clumsiness	+	−	+	−	−	+	−	−	+	+	−	No candidate variants	
P34, M	11	Aff sib (P33)	Upper limb clumsiness	+	+	+	+	+	+	−	−	+	+	−	No candidate variants	
P35, F	20	Mother has hypotonia	Gait disturbance	+	+	+	−	+	−	−	−	−	+	−	No candidate variants	

M = male; F = female; Hom = homozygous; Het = heterozygous; ‘+’ = present; ‘-’ = absent, not applicable (test not carried out); sib = sibling; aff = affected; N = normal (all results normal/negative).

^s^Segregation analysis performed in the family.

Two have been reported previously (Pyle *et al.*, 2012, 2013).

### Molecular genetics and bioinformatics

Blood genomic DNA was fragmented, exome enriched and sequenced (Illumina TruSeq™ 62 Mb and HiSeq 2000, 100 bp paired-end reads). Coverage data are summarized in Supplementary Tables 1 and 2. In-house bioinformatic analysis included alignment to UCSC hg19, using Burrows-Wheeler Aligner; duplicate removal (Picard v1.85) and variant detection (Varscan v2.2) (Koboldt *et al.*, 2009), Dindel v1.01 (Albers *et al.*, 2011). Further analysis was performed on variants with a minor allele frequency <0.01 in several databases: dbSNP137, 1000 Genomes (April 2012 data release), the National Heart, Lung and Blood Institute (NIH) Exome Sequencing Project (ESP) 6500 exomes, and 286 unrelated in-house controls. Rare homozygous and compound heterozygous variants were defined, and protein altering and/or putative ‘disease causing’ mutations, along with their functional annotation, were identified using ANNOVAR (Wang *et al.*, 2010). Putative pathogenic variants were confirmed by Sanger sequencing using custom-designed primers (http://frodo.wi.mit.edu) (ABI BigDye® v3.1 3130xl Genetic Analyzer, Life Technologies). Comparative genomic hybridization was performed in presumed recessive cases where a single likely pathogenic allele was found in a strong candidate gene. Quantitative pyrosequencing was used to determine the proportion of mutated alleles in given tissues (Pyromark v2.0, Qiagen; Supplementary Table 3).

### Variant classification

Variants were defined using *a priori* criteria: (i) confirmed pathogenic—a variant previously shown to be pathogenic, or in a known ataxia disease gene where the variant was predicted to affect protein structure or function, and segregated with at least one additional affected family member; (ii) possible pathogenic variants—variant in a known ataxia gene and predicted to affect protein function but not fulfilling all of the above criteria; and (iii) variants of uncertain significance or no candidate variants found.

## Results

**Figure 1:**
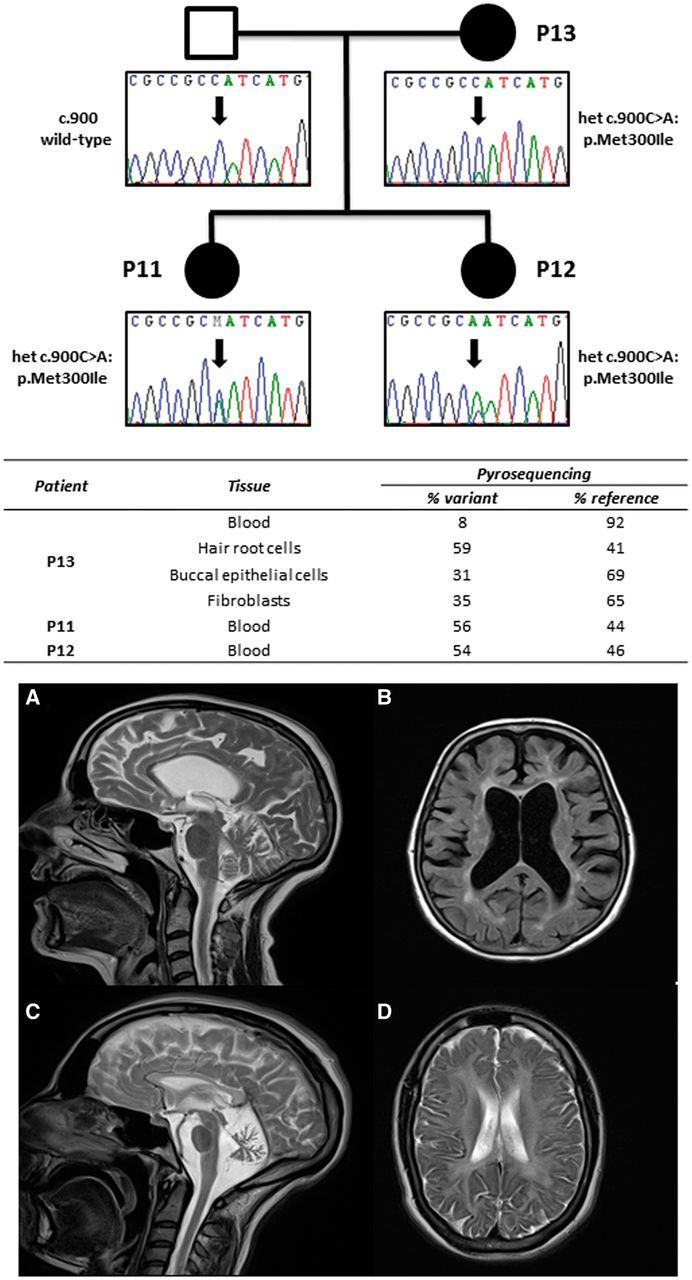
***De novo TUBB4A *mutation in Patients P11, 12 and 13.** *Top*: Segregation of *TUBB4A *c.900C>A (p.Met300Ile) mutation in Patient P13 (mother), and her two daughters. Confirmatory Sanger sequencing and pyrosequencing in different tissues from the mother showing tissue mosaicism in the mother for the presumed *de novo *dominant allele. *Middle*: Pyrosequencing results of this mutation in different tissues. *Bottom*: Brain MRI from Patient P13, the mother (**A** and **B**) and daughter Patient P12 (**C** and **D**). (**A** and **B**) T_2_ and T_1_ images showing generalized atrophy and periventricular high signal. (**C** and **D**) T_2_ images showing marked cerebellar atrophy and diffuse hypomyelination.

Confirmed pathogenic variants were found in 9/22 probands (41%) (Table 1 and Supplementary Table 4). Three families had novel compound *SACS *mutations, each found in two affected siblings, including a full deletion of *SACS *detected from exome coverage (Pyle *et al.*, 2012, 2013). Known compound heterozygous *SPG7* mutations were found in three affected individuals from one family with no spasticity (Casari *et al.*, 1998). Two siblings presenting with adult-onset ataxia had compound heterozygous mutations in *NPC1*, confirmed by subsequent oxysterol analysis (Supplementary Table 5) (Carstea *et al.*, 1997). Likely *de novo *dominant *TUBB4A *mutations were found in two families (Simons *et al.*, 2013). One family showed varying degrees of mosaicism in the mildly affected mother and heterozygosity in the severely affected offspring (Fig. 1 and Supplementary Table 6). A *de novo* dominant *SLC1A3 *mutation segregated with ataxia in three members of a family (de Vries *et al.*, 2009). Finally, a dominant *KCNC3 *mutation previously described in a large Filipino kindred and three European index cases (Waters *et al.*, 2006; Subramony *et al.*, 2013) segregated with ataxia in four members of a three-generation autosomal dominant pedigree.

**Figure 2:**
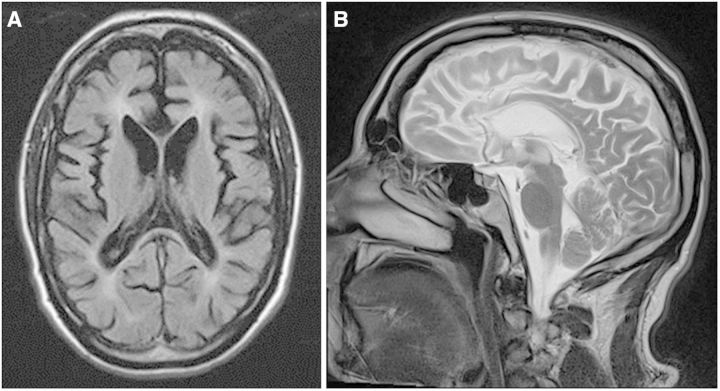
**T_1_ axial (A) and T_2_ sagittal (B) MRI in Patient P19. **The MRI shows global cerebral atrophy with relative sparing of the occipital lobes and cerebellum, and marked atrophy of the corpus callosum previously described in patients with mutations in *ZFYVE26 *(SPG15) (Goizet *et al.*, 2009).

Possible pathogenic variants were identified in 5/22 probands (23%), including compound heterozygous *FASTKD2 *variants (c.149A>G:p.Lys50Arg, which is highly conserved; and c.-66A>G predicted to affect exon 1 splicing) (Ghezzi *et al.*, 2008). One proband had *de novo *compound heterozygous mutations in *ZFYVE26 *(SPG15) (Fig. 2) (Hanein *et al.*, 2008). A predicted splice site mutation, c.805-2A>G (Supplementary Fig. 1), was detected in three members of an autosomal dominant pedigree in the previously described gene *ZFYVE27* (Mannan *et al.*, 2006). Previously described compound heterozygous *WFS1 *mutations (p.Lys193Gln and p.Arg456His) were identified in one family (Cryns *et al.*, 2003). Detailed clinical data and the results of segregation analyses are included in the Supplementary material.

We were unable to confidently identify likely candidates in 10/22 probands but present potential candidate genes that may be recognized by others.

## Discussion

In this study whole exome sequencing identified confirmed pathogenic variants in 9/22 families with unexplained ataxia, with a similar diagnostic yield in both young (<20 years of age) 4/9 (44%) and older-onset (>20 years of age) 5/9 (56%) patients. Likely pathogenic variants were found in 5/22 probands, again with a similar diagnostic yield in both young (<20 years of age) 2/5 (40%) and older-onset (>20 years of age) 3/5 (60%) cases. The likely genetic cause was identified in 2/4 (50%) of adult-onset sporadic cases, and one gene was implicated in 3/6 (50%) of early-onset presumed recessive cases. Taken together these account for the likely molecular diagnosis in 64% of our families. These findings contrast with those of a targeted next generation sequencing approach, which identified the cause in 18% of patients in a similar study cohort (Nemeth *et al.*, 2013). Greater genome coverage provides the likely explanation for the higher diagnostic yield reported here, underscored by our observation that some of the definite mutations were found in genes known to cause ataxia, but not always considered ‘ataxia genes’, and thus not included on some multi-gene panels (Nemeth *et al.*, 2013). The extra coverage had the greatest impact on the diagnosis of adult-onset patients with recessive ataxia—a disorder generally considered to be of childhood onset. Our findings are endorsed by a recent study in children (Sawyer *et al.*, 2014), but unexpectedly show the potential impact of exome sequencing in patients with suspected inherited ataxia presenting at any age.

With 11 genes implicated in 14 families, and no recurrent mutations in the same gene, our observations reaffirm the known genetic heterogeneity of inherited ataxia in an outbred European population. Similar genetic heterogeneity was also noted in 46 patients with sporadic and familial cerebellar ataxia studied with exome sequencing (Fogel *at al*., 2014). Of the 16 patients with a confirmed diagnosis, only one of the disease genes identified by Fogel *et al.* (2014) was also identified in our patients (*SPG7*), and only two of the genes implicated in the remainder (*WFS1*, *ZFYVE26*) were also implicated in the patients we describe here (Table 1). In both our study and the work of Fogel *et al.*, (2014), the non-targeted exome sequencing approach increased the likelihood of detecting causal variants, particularly those in newly described disease genes, or exceptionally rare disease genes not found on targeted capture arrays. Although we did not directly compare exome sequencing to an ‘ataxia multi-gene panel’, our results show that exome sequencing is highly likely to have a greater diagnostic yield. In one of the largest panels produced to date, Nemeth *et al.* (2013) studied 117 known and putative ataxia genes. Eighty-nine per cent of the coding sequence of these genes was covered >20-fold using our exome sequencing approach, so it is highly likely that we would have detected the mutations found in their cohort. On the other hand, only 29% of the disease genes that we identified were included in the multi-gene panel of Nemeth *et al.* (2013), including genes not previously considered to be ‘ataxia genes’, such as *SPG7*. Moreover, there may be limited overlap between different multi-gene panels, which are defined by specific laboratories reflecting ataxia in a given population, and based on ataxia genes known at that time. Sequencing the entire exome is likely to address these concerns. Given that exome sequencing costs are approximately the same as a single candidate gene test in most diagnostic laboratories, exome sequencing provides a rapid and cost effective means of reaching a diagnosis in a group of patients that have been notoriously difficult to diagnose at the molecular level.

A further advantage of the exome-based approach is the identification of known disease genes in a different clinical context, broadening the phenotype. With the potential for false positives, we also sought corroborative phenotypic or biochemical data when we detected a variant in an unexpected gene. This approach confirmed the diagnosis of Niemann Pick type C in two siblings with adult-onset ataxia, but lacking the characteristic eye movement disorder seen in childhood (Patients P15 and P16), and also prompted careful review of neuroimaging, which confirmed the thin corpus callosum characteristic of *ZFYVE26*/SPG15 (Patient P19, Fig. 2) (Goizet *et al.*, 2009). In these patients, exome sequencing highlighted likely causal variants, which were substantiated by subsequent clinical and biochemical studies. On the other hand, we observed an adult-onset for ataxic disorders generally considered to present in childhood (*SACS*), and atypical clinical presentations, such as sacsinopathy presenting with a Charcot–Marie–Tooth phenotype. Likewise, although ataxia has been described as a presenting feature of *SPG7*, the absence of spasticity was thought to reduce the likelihood of a positive *SPG7 *result, moving this specific gene test down the priority list. These observations demonstrate the importance of iterating between the clinical and genetic data to maximize the potential of large-scale sequencing, not only providing a diagnosis for a specific family, but also advancing our understanding of the phenotypic spectrum of specific disease genes.

We did not observe many of the rare autosomal recessive forms of ataxia observed in a similar sized UK cohort (Nemeth *et al.*, 2013), reflecting the genetic heterogeneity of inherited ataxia. We suspect that the extreme genetic heterogeneity has prevented us from identifying any recurrent novel ataxia genes with any degree of confidence. However, publishing our findings will hopefully allow others to refer to the variant data in our undiagnosed cases, and thus reach a final conclusion.

The use of exome sequencing in neurogenetic disorders is endorsed by recent findings in 24 cases (15 families) with undiagnosed suspected inherited polyneuropathies (Charcot–Marie–Tooth disease), where pathogenic mutations were found in five probands and possible causal mutations in three (Klein *et al.*, 2014), giving a total yield of 53%. An even higher yield may be possible for presumed autosomal recessive disorders when mother-father-affected child trios are sequenced, given the recent findings in 55 families with hereditary spastic paraplegia where the likely diagnosis was defined in 75% (Novarino *et al.*, 2014). However, identifying trios is challenging for adult-onset cases where parental samples are not readily available, and the advantage of trios is not so apparent for autosomal dominant disorders, which account for 44% of ataxia patients we describe here.

Although the whole exome approach provides a major advance in likely diagnostic yield in suspected inherited ataxia, the approach may uncover unexpected findings, such as mutations in genes known to cause familial cancer syndromes, or a different neurodegenerative disease such as familial Alzheimer’s disease. These incidental findings are rare, and were not discovered in this study, but must be considered when taking informed consent for exome studies (Bamshad *et al.*, 2011). It should also be borne in mind that our study was based on only 22 probands from one geographic region. These patients were from a largely outbred stable population of white European extraction, and the spectrum of different disease-causing alleles and genes may differ in other parts of the world, particularly if there is evidence of a founder effect. In these contexts a more focused sequencing approach may be more appropriate. Our study also had wide entry criteria, reflecting routine clinical practice. Although it is reassuring that exome sequencing appeared to be effective across this clinical spectrum, larger studies may show that it is more or less effective in specific clinical subgroups. Finally, we were unable to identify the likely genetic basis of the ataxia in 36% families. Exome sequencing the parents may reveal the discordant heterozygous variant responsible for the disorder that is otherwise challenging to identify. This is particularly relevant for congenital or childhood-onset ataxias (Ohba *et al.*, 2013), but many *de novo* dominant mutations can also cause adult-onset ataxia, as shown here (Fig. 1). It remains possible that some of the later-onset cases are not primarily genetically determined; however, given the relevant family history in most, there are also likely to be technical explanations. Exome capture does not provide complete coverage of all coding regions of the genome, particularly those with GC-rich regions. In addition, large genomic rearrangements and trinucleotide repeat sequences are not reliably detected from exome-capture data. It is therefore possible that some of the undiagnosed cases have pathogenic trinucleotide expansions not routinely tested in our region, including SCA10, 12 and 31, although these are very rare in our experience. Finally, it is likely that some causal variants will reside within non-coding regulatory regions. Some of these issues will be resolved by whole genome sequencing, although not without substantial additional cost and bioinformatics complexity.
